# Assessing organisational readiness for change: use of diagnostic analysis prior to the implementation of a multidisciplinary assessment for acute stroke care

**DOI:** 10.1186/1748-5908-2-21

**Published:** 2007-07-14

**Authors:** Sharon Hamilton, Susan McLaren, Anne Mulhall

**Affiliations:** 1 Director, Centre for Health and Social Evaluation (CHASE), University of Teesside. Parkside West, Middlesbrough, Tees Valley, TS1 3BA, UK; 2 Director, Centre for Leadership and Practice Innovation, Faculty of Health and Social Care, London South Bank University, London, SE10AA,UK; 3 Independent Consultant, Rectory Road, Ashmanhaugh, Norfolk, NR12 8YP, UK

## Abstract

**Background:**

Achieving evidence-based practice in health care is integral to the drive for quality improvement in the National Health Service in the UK. Encapsulated within this policy agenda are challenges inherent in leading and managing organisational change. Not least of these is the need to change the behaviours of individuals and groups in order to embed new practices. Such changes are set within a context of organisational culture that can present a number of barriers and facilitators to change. Diagnostic analysis has been recommended as a precursor to the implementation of change to enable such barriers and facilitators to be identified and a targeted implementation strategy developed. Although diagnostic analysis is recommended, there is a paucity of advice on appropriate methods to use. This paper addresses the paucity and builds on previous work by recommending a mixed method approach to diagnostic analysis comprising both quantitative and qualitative data.

**Methods:**

Twenty staff members with strategic accountability for stroke care were purposively sampled to take part in semi-structured interviews. Six recently discharged patients were also interviewed. Focus groups were conducted with one group of registered ward-based nurses (n = 5) and three specialist registrars (n = 3) purposively selected for their interest in stroke care. All professional staff on the study wards were sent the Team Climate Inventory questionnaire (n = 206). This elicited a response rate of 72% (n = 148).

**Results:**

A number of facilitators for change were identified, including stakeholder support, organisational commitment to education, strong team climate in some teams, exemplars of past successful organisational change, and positive working environments. A number of barriers were also identified, including: unidisciplinary assessment/recording practices, varying in structure and evidence-base; weak team climate in some teams; negative exemplars of organisational change; and uncertainty created by impending organisational merger.

**Conclusion:**

This study built on previous research by proposing a mixed method approach for diagnostic analysis. The combination of qualitative and quantitative data were able to capture multiple perspectives on barriers and facilitators to change. These data informed the tailoring of the implementation strategy to the specific needs of the Trust.

## Background

Achieving evidence-based practice in health and social care is integral to the drive for quality improvement in the NHS [1]. Encapsulated within this policy agenda are challenges inherent in leading and managing organisational change. Not least of these is the need to change the behaviours of individuals and groups in order to embed new practices. Such changes are set within a context of organisational culture, resources, economic and political factors that can create uncertainty [2]. A recent systematic review by Shaw *et al*. [3] emphasised that while some strategies to change professional behaviour are successful, others may not exert a positive impact due to barriers operating in local settings, which can vary over time. Such barriers operate not only at the level of the individual but also in conjunction with the social and organisational contexts of care provision [4,5]. However, facilitators for organisational change can also be present, and it is essential to identify these prior to implementing new practices, for example through evidence-based standards and guidelines, opinion leadership, education or other strategies likely to improve the uptake of an innovation [6,7]. Strategies which are designed or 'tailored' to overcome barriers [5] and maximise the impact of facilitators are most likely to embed change [8], although the evidence from the small number of studies that have addressed this is not conclusive.

Diagnostic analysis requires gathering information prior to the implementation of change, and is designed to identify the complexities (barriers and facilitators) within an organisation that may frustrate or facilitate the uptake of change [9]. Theoretical models underpinning change are useful for guiding the design of diagnostic analysis. For example, elements of diagnosis are evident in some stage models of change, notably the preliminary stages of force field analysis [10]; social marketing [11], and the 'precede/proceed' model of Green and Kreuter [12]. These models are based on the assumption that change is linear and is amenable to rational planning. However, it has been argued that this is not appropriate to the healthcare environment, as change is more likely to be disorderly, dynamic, and uncertain as a result of the complexities of organisational life [13]. Elements of diagnostic analysis are also evident in organisational development theory. The underlying assumptions of this are that change can and should be planned, and further, it emphasises the need to involve key stakeholders in identifying barriers to change [14-16].

Contextualist approaches challenge rational-linear stage models (such as Lewin [10]), and emphasise the need to consider internal organisational factors (past history of change, culture, social networks, political and economic environment) in assessments of readiness to implement an innovation[17].

These theoretical models inform the planning of change, however Shaw *et al*. [5] have commented that more research is needed to identify and overcome barriers to implementation. Comparatively few studies that have employed diagnostic analysis have discussed the methodological approaches used in any depth. Exceptions include Turrell [18], who used semi-structured interviews and a documentary analysis to identify internal and external factors that could impact on the production of nursing practice guideline documentation. A systematic review by Davis *et al*. [19] concluded that interventions were most likely to change medical practice when informed by a preliminary analysis of educational needs and barriers to change identified using survey methods. In the South Thames Evidence-Based Practice Project (STEP), a range of formal and informal diagnostic methods was used to elicit views and opinions of key stakeholders (patients and staff) encompassing assessments of work environment, semi-structured interviews, focus groups, questionnaires, documentary analysis (case-notes, Trust strategies, policies), direct observation, and structured reflection by change agents using field diaries [8,19-21]. In contrast, Newman *et al*. [22] conducted a rapid organisational appraisal utilising formal and informal interviews, focus groups, observation of meetings and clinical practice to identify organisational barriers for change.

Wensing and Grol [23] emphasised the value of diagnostic analysis as a precursor to change and highlighting a number of examples from previous studies. They recommended the use of a mixed method approach which could include interviews, observation and survey methods. The diagnostic analysis reported here builds on these recommendations by including an analysis of publicly available corporate documents. Such documents make a useful starting point for highlighting gaps between these aspirations and their operationalisation as perceived by staff and patients.

In summary, the design of this diagnostic analysis was influenced by a range of theories, including organisational development theory [24] which emphasised the need to plan change and involve key stakeholders in the earliest stages to identify barriers to change. Previous studies [8,19-22] utilising diagnostic analysis had identified a mixed methods approach to be of benefit in planning for successful change, for example, in relation to implementation of evidence-based standards and guidelines designed to improve the quality of practice and service. Later stages of this study were intended to implement guidelines for multidisciplinary stroke assessment [25].

Specific aims were to identify barriers and facilitators for change to multidisciplinary stroke assessment and to utilise the information obtained to inform a change management approach tailored to local context. Specific objectives were to identify past experiences of change in the Trust, to evaluate the extent and nature of multidisciplinary team working and to secure early ownership of the project from key stakeholders (health professionals, managers, and patients). A methodological objective was to test the usefulness of a specific combination of methods for undertaking diagnostic analysis.

## Methods

### Setting

The study was conducted in an acute NHS Trust (600 beds) over a six-month period and formed stage one of a larger study, the aim of which was to evaluate the implementation of a multifaceted strategy for implementing change in stroke assessment. The Trust did not have a stroke unit and patients were admitted to one of nine medical/care of the elderly wards. At the time, the Trust was about to go through a merger process with a neighbouring Trust and this was causing much uncertainty and organisational instability.

### Sampling framework and methods

The study received ethical approval from the Local Research Ethics Committee. Four data collection methods were used: documentary analysis of six publicly available Trust documents; two focus groups totalling eight ward-based professional staff; interviews with twenty senior professional staff and six recently discharge patients; and a questionnaire to all professional staff involved with stroke care at the Trust (n = 206) producing a 72% response rate (n = 148).

The mixed method approach was a particular strength of the diagnostic analysis. It provided the tools to capture multiple realities and the individual perspectives that made up the social situation of stroke care at the Trust. The notion of multiple realities was important in this study, as it was vital that the views of the more powerful and vocal stakeholders did not dominate so that those who might feel less confident could make a contribution. Further, it had the potential to add breadth, rigor and credibility [26]. Mixed method designs combine the benefits of qualitative methods (*e.g*., capturing the unexpected, richness in explanation, and supporting participants to define their contributions) and quantitative methods (ability to compile and summarise large amounts of information) to create a holistic approach to data collection and analysis [26].

Documentary analysis was conducted on six authentic, verifiable Trust documents in the public domain to obtain evidence on strategies supporting evidence-based practice, clinical effectiveness and quality outcome measures used by a range of professional groups. Furthermore, decision making structures in the organisation, policies for staff development, multiprofessional working, and resources available to support the implementation of practice change were identified together with networks for the dissemination of information. The range of documents analysed included annual reports, research and development reports, Trust profile, nursing and midwifery strategy, and five-year vision for health service delivery. Documentary data were abstracted and analysed thematically using a structured framework [27,28].

Interviews and focus groups were used to elicit the views and experiences of professional staff who would be affected by changes in stroke assessment and recording. Twenty staff with strategic or operational accountability for aspects of stroke care were purposively sampled (ward managers, executives, medical consultants, service managers, senior dieticians, and senior physiotherapists) to take part in semi-structured interviews. An interview topic guide focused on organisational culture, organisational history, stroke assessment, and multiprofessional working. The same topic guide was used with focus groups. Focus groups were conducted with one group of qualified ward nurses (n = 5) and three specialist registrars (n = 3).

Interviews were also conducted with six patients one month after discharge following an acute stroke. The interview schedule was structured, containing sequential questions exploring experiences from the time of admission to discharge, focussing on satisfaction with care, activities of daily living, physical problems encountered, and the awareness of these problems in the assessment.

All interviews and focus groups were tape-recorded and transcribed with the permission of the participants, independently checked for reliability of transcription by a senior academic, and analysed thematically using a structured framework [27]. An additional check of validity came from returning transcripts to respondents for their agreement that it accurately represented their views [29,30]. A further check would have been to return the analysis to respondents for comments [31], however, assurances of confidentiality had been given that would have been violated by respondents having access to the comments of other respondents. Content validity for each of the data collection tools was informed by the literature review, and face validity was strengthened by the involvement of a senior academic with expertise in stroke care.

### Team climate inventory (TCI)

This multidimensional measure of workgroup climate developed and validated by Anderson and West [32] was used to evaluate existing teamwork practices across the range of professional disciplines responsible for stroke assessment and recording. The TCI comprises a 44-item questionnaire, with responses graded on a five point Likert scale across indicators of team function relating to communication, innovation, objectives, and task style. TCI questionnaires were distributed via the internal post to the total population (n = 206) of nurses, therapists and medical staff working on the 9 study wards. This elicited an overall response rate of 72% (n = 148) following reminders. Respondents were asked to indicate, in their own words on the front of the questionnaire, the team to which they belonged. Respondents indicated affiliation with nine teams: nursing (seven ward teams, n = 105); medicine (one team operating across all wards, n = 27); therapists (physiotherapists, occupational therapists, dieticians, speech and language therapists (one team operating across all wards, n = 16). Responses were analysed using TCI software to generate 'STEN' (Standardised Ten) scores across the areas of participative safety, support for innovation, vision, and task orientation. Extensive validation of the TCI questionnaire had previously been undertaken. Importantly for the study reported here, tests for predictive validity regarding innovativeness had been conducted with NHS teams [32].

## Results

Documentary analysis: five themes were identified: evidence-based practice and clinical effectiveness; management approaches and decision making structures; staff development and training; multiprofessional working; and support for the project.

### Theme one: evidence-based practice and clinical effectiveness

The organisation supported evidence-based practice and research and development initiatives, although a process for implementing such innovations as part of clinical governance procedures was not identified.

'Clinical effectiveness and clinical governance are key pillars on which the government wants health care to be built. This Trust already has a reputation for quality.' (Trust Annual Report.)

'R&D is the foundation of evidence-based practice, and should be the basis for planning and delivering clinical care.' (R&D Annual Report)

An encouraging finding was the commitment to the provision of training in critical appraisal skills and development of nursing outcome indicators.

'The Trust will encourage training in R&D methodology and critical appraisal of research findings to contribute to evidence-based medicine'. (R&D Annual Report)

'... the development of valid nursing outcome indicators in collaboration with clinical audit, research and development. (Nursing and Midwifery Strategy).

### Theme two: management approaches and decision making structures

The Trust Profile provided an overview of a clear and unambiguous decision making structure, with a commitment to participative decision making and multidisciplinary team work.

'The general approach taken to the management of the Trust is based on the following principles: maximum devolution of authority [and]multidisciplinary teams at all levels'. (Trust Profile)

Multidisciplinary teams were responsible for the management of ten service areas. Medical professionals were the leaders in nine teams. However, it was not clear how multidisciplinary teamwork operated at ward level.

### Theme three: staff development and training

Although a strong emphasis on staff training and development was identified in all the documents analysed, exemplars of training and educational achievements were not presented. Explicit commitment was given:

'... to be a teaching, learning and research organisation' (Trust Profile)

A high profile was given to education as part of continuing professional development, and explicit links were made between education and improved patient care. Education was also presented as creating a positive environment that would improve staff recruitment.

'Education too is crucial to the delivery of patient quality care. All employees are encouraged to increase their knowledge and, as a result, achieve practical benefits' (Annual Report)

'... a progressive employer, enabling staff to realise their full potential and being an organisation in which people wish to work'. (Trust Profile)

While there was clear support for education and training generally, nursing leadership, research, and supervision were also emphasised in terms of commitment.

'... clinical and professional leadership, research and supervision'(Nursing and Midwifery Strategy)

### Theme four: multiprofessional working

The decision making structure of the Trust highlighted the adoption of a multidisciplinary approach, yet little operational evidence was found of this commitment, with reference to only one initiative.

'... a multidisciplinary surgical procedure review committee will be established to assist the rationalisation of procedures based on outcome measures of success in accordance with a certification process developed by the Royal College of Surgeons' (Five Year Vision for Health Services)

### Theme five: support for the project

It was important that the Trust demonstrated support for the project so that staff understood that the changes would be embedded into Trust business. Overt Trust support was also important for underpinning the relationship between the project leader and professional teams who would be implementing the change, as the project leader did not have any line management responsibility for the teams involved. The project leader had to use influencing skills to achieve changes in practice. Influence was needed at all levels in participating teams, including ward-based staff and senior managers. The project leader worked through formal structures, such as directorate meetings and Trust board members, and also through informal opportunities that occurred from visiting the wards every day. Teams were free to choose how they implemented the change. For example, ward sisters monitored the use of the multidisciplinary recording system in some wards, while medical consultants generally did not supervise its use by their junior medical staff.

Trust support was clear in terms of funding and the appointment of the researcher as cited in three documents. Furthermore, the Trust was moving towards implementing the unique electronic patient record and expectations were that the project would link with this.

'Nursing documentation/records will be completely reviewed and updated ready for incorporation into the "unique medical record"' (Nursing and Midwifery Strategy).

### Interviews and focus groups (staff)

Four themes were identified: stroke assessment and recording, stroke services, the Trust as an organisation, and past history of change at the Trust.

### Theme one: stroke assessment and recording

The assessment of stroke patients was fragmented, unidisciplinary, separately documented, and lacked cohesion. Furthermore, the use of evidence-based, validated assessment tools varied across disciplines. Exemplars included the Waterlow Scale [33] and standardised swallow assessment [34], both of which were used by nurses in conjunction with a series of questions based on the Roper, Logan & Tierney nursing model [35]. Medical staff used an assessment model based on anatomical and physiological systems together with three validated assessments: the Glasgow Coma Scale [36]; the Abbreviated Mental Test Score [37] and the Barthel Index [38]. Not all physiotherapists routinely used assessment tools, but in one area, a modified Rivermead Mobility Index [39] had been adopted. In speech and language therapy, routine use of the Frenchay Dysarthria Assessment [40]; the Frenchay Aphasia Screening Test [41] and the Psycholinguistic Assessment of Language in Aphasia [42] were reported.

All respondents described how each discipline undertook an individual assessment of stroke patients and documented the results in separate records. Only the medical and nursing assessments were accessible to all professional groups on the ward.

'It's often difficult for us nurses because we don't know what's happened to them (the patient) in physio or OT, there's nothing written for us to read, they (the physios and OTs) do their assessments in the gym and we don't get to see it' (Nurse – interview)

Some therapists, in addition to maintaining separate, full assessment records, also recorded abridged versions in the medical and nursing notes, omitting technical details. Some professionals reported that the current approach to stroke assessment did not have any gaps, but nurses were concerned that their assessments did not contain enough information to inform the care plan.

'... that's a big gap, it's about how we as a professional organisation enable nurses to link the two together, with assessment informing care planning' (Manager – interview)

Similarly, lack of written detail in the medical assessment relating to functional assessment was also identified as a gap.

'Doctors use a structured approach to assessment but sometimes don't write down enough detail, for example, right hemiplegia doesn't tell you much' (Doctor – focus group)

### Theme two: stroke services

Many respondents acknowledged that comprehensive multidisciplinary working did not occur, and identified multidisciplinary team meetings that varied functionally as the main focus.

'The level of multidisciplinary working varies, but the multidisciplinary team meetings are the focus of multidisciplinary working on the wards, and they work better on the elderly care wards' (Allied Health Professional – interview)

Liaison was highlighted as part of multidisciplinary working, with mixed views expressed about its efficacy. The diverse geographical spread of wards to which stroke patients were admitted, the numbers of different nurses involved, and lack of a stroke unit made liaison difficult.

'Its hard keeping track of patients, there's no dedicated stroke unit, that's the problem, if all stroke patients were in one place it would be so much easier for all staff to liaise, therapists would be there on the unit' (Manager – interview)

Despite the lack of a stroke unit, perceptions varied of the quality of service delivered.

'The service varies, in some areas it's not very good, within some areas there are probably some areas of good practice as well, but I would say that overall it is about average' (Manager – interview)

### Theme three: the Trust as an organisation

Overall, participants identified many strengths, most commonly the positive working environment and management structures which were flat hierarchies, with devolved decision making.

'There aren't too many layers of management, the Trust has a fairly flat hierarchical structure' (Doctor – interview).

'I think people feel involved in decisions, the Trust has made good attempts to devolve decision making' (Manager – interview)

Limitations were also evident. Notably, the devolved management structure often led to communication problems. Furthermore, shortages of nursing staff were evident, however this was part of a wider national problem. A lack of qualified nurses had led to use of agency and bank nurses. Different agency nurses worked on the wards each day, which led to lack of continuity.

'Sometimes I despair, I don't know why we can't have the same agency nurse if she's available, it means that each shift I have to start again, tell her all the things about how we do things on the ward' (Nurse – focus group)

### Theme four: the organisational history of change

The general consensus from participants was that the Trust responded positively to change and that this was a constant feature of working in the NHS.

'... it's (change) so constant now isn't it, if you can't cope with it you're gone, its very hard, you're still reeling from the last one when the next one comes along' (Manager – interview)

Many exemplars of well-managed change were cited, including a recent rationalisation of services, introduction of pharmacy stations on wards, introduction of swallow screening and extended roles for nurses. Key characteristics of these changes were good communication, planning, involvement of staff, and training provided prior to implementation. Exemplars of less well-managed change, including changes to catering services, a change in the way ward-based nursing was organised, changes from mixed to single-sex wards. These changes were described as poorly communicated, brought in too quickly and lacking adequate staff consultation and preparation.

### Patient interviews

All patients reported their care to be good to excellent and five patients said they would recommended the care and treatment to others. A need for improvements in access to a wheelchair and the poor quality of food were noted by two patients. Three patients and one caregiver were satisfied with the information provided on admission. Three patients were not provided with discharge information. Four patients could recall specific positive aspects of treatment by therapists. All who were referred to therapists were satisfied with their treatment, and five patients generally were satisfied with their recovery. In four cases, occupational therapy was restricted to a home assessment due to staff shortages.

### Team climate inventory

Important findings from the Team Climate Inventory questionnaire (Table 1) were variability in teamwork across the professional groups. Scores of eight or above indicated excellent team working; scores between four and seven indicated room for improvement; scores of less than four indicated low levels of teamwork.

**Table 1:** Results of team climate inventory: STEN scores for each team

**Items and Sub-scales from TCI**	**Nursing Teams**							**Medical**	*Therapy
**Team Number**	**1**	**2**	**3**	**4**	**5**	**6**	**7**	**8**	**9**
***Participative Safety***									
Information sharing	10	5	8	6	6	7	3	5	6
Safety	7	4	6	7	7	6	3	7	10
Influence	8	5	5	6	8	5	2	4	10
Interaction frequency	8	5	6	5	8	7	3	3	9
**Overall STEN for sub-scale**	8	5	6	6	7	6	3	4	9
***Support for Innovation***									
Articulated support	9	6	10	8	6	8	4	8	10
Enacted support	10	7	10	8	6	8	4	6	6
**Overall STEN for sub-scale**	9	6	10	8	6	8	4	7	8
***Vision***									
Clarity	9	5	8	6	7	6	4	3	9
Perceived value	9	4	9	7	8	9	4	9	10
Sharedness	6	3	6	4	6	4	4	2	10
Attainability	7	5	8	6	7	8	5	5	7
**Overall STEN for sub-scale**	8	4	8	6	7	7	4	4	10
***Task Orientation***									
Excellence	8	6	8	8	8	7	4	2	8
Appraisal	6	5	6	5	6	4	3	1	6
Ideation	8	5	8	7	8	7	6	6	8
**Overall STEN for sub-scale**	**7**	**5**	**8**	**7**	**7**	**5**	**4**	**2**	**7**

*Therapy team = occupational therapists, physiotherapists, speech and language therapists, and dieticians.

Scores 8 or above (excellent team working); scores between 4 and 7 (room for improvement in team working); scores less than 4 (low levels of team working).

The therapy team scores ranged from six to ten, with nine subscale items scoring more than eight (indicating excellent team working) and scores on only four items (range six to seven) suggested room for improvement. In contrast, the medical team subscores ranged between one and nine, with only two items scoring more than eight and six items less than four. Low scores on 'interaction frequency', 'clarity', 'sharedness', 'appraisal', and 'excellence in task orientation' were matters of concern. Nursing team two (range on subscores three to seven; one item scored above eight) and seven (range on subscores tow to six; no item scored above eight) demonstrated the weakest teamwork. All aspects of 'participative safety' in team seven produced low scores, and the low score for 'sharedness' in team two were matters of concern. In contrast, nursing teams one and three were the highest scoring, the former with nine items scoring above eight, and the latter with eight items scoring above eight.

### Developing a tailored strategy for local change

A summary of the barriers and facilitators for change in stroke assessment practices together with their implications as identified from this diagnostic assessment are summarised in Table 2. Key issues related to communication and perception of the change, workforce issues including severe shortage of professional staff, unidisciplinary assessment and working practices, and a lack of organised stroke care in the organisation.

**Table 2:** Diagnostic findings: implications

**Organisational Factors**		
**Barriers**	**Facilitators**	**Implications**

Communication problems, lack of staff consultation, preparation and ownership associated with past history of poorly managed change	Good communication, planning and training provision associated with past history of well managed change.	Build on previous success. Clarify lines of communication; establish local ownership; use training intervention to benefit staff skills related to changes.
Uncertainty relating to a potential Trust merger; possible negative impact on management, staff capacity and work environment	Positive staff views of the work environment and management structure. Local project support explicit.	Strengthen teamwork; set up project steering group with influential support; work across organisational boundaries.
Nursing workforce shortages; use of agency staff leading to potential discontinuity in assessment and care planning.	Core of stable senior staff: median service of interviewees 10 years.	Ensure agency staff included in outreach training to implement assessments; flexible scheduling of training to maximise attendance.
Processes for implementing innovations not clear in organisational strategy.	Strategic commitment to clinical effectiveness, multidisciplinary working, *evidence-based *medicine, education of staff.	Develop strategy using recognised clinical effectiveness methods, education/training, multidisciplinary approaches

**Teamwork Factors**		

Team work less well developed in medicine and some areas of nursing	Team work strongly developed in therapies and some areas of nursing; positive role models exist.	Create positive focus and environment for team work within strategy eg shared training; outreach necessary in areas of weak team work and staff shortages.
Team concept; unidisciplinary Negative views of multidisciplinary ward meetings and efficacy of liaison. Service teams and ward meetings largely medically led.	Positive views of multidisciplinary ward meetings and efficacy of liaison. Leadership potential evident within therapy and some nursing teams	Professional representatives/champions needed to provide leadership on equal basis to drive change

**Stroke Assessment Factors**		

Assessments unidisciplinary, fragmented, variable evidence-base, and using separate recording systems. Negative experiences of patients on discharge information provided	Local commitment to developing evidence-based practice. Need for assessment project supported by Trust. Positive views of patients on assessment and care provided	Utilise evidence-based guidelines for assessment and recording. Mechanisms for critical appraisal to be set up. Guidelines for discharge information needed

## Discussion

A need exists to identify and overcome local barriers before the implementation of new practices in organisational settings can be implemented [5]. A diagnostic analysis was recommended for this purpose [9]. However, a limited range of approaches or methods had been proposed. In this study, a combination of qualitative and quantitative methods were chosen, including documentary analysis, interviews, and focus groups, because these had previously been used successfully in the STEP project [8], as well as by Newman [22] and Turrill [18]. Data from this combination of methods identified a complex mix of organisational, teamwork, and specific assessment-related factors as barriers and facilitators for change.

Organisational factors are acknowledged to be influential in determining the outcome of practice change [43]. Pettigrew has emphasised the importance of investigating the past history of organisational change in planning future developments [17]. In this organisation, examples of successful change were characterised by effective planning and communication with key stakeholders, and the provision of appropriate training when new skills were required. Negative experiences were associated with poor communication, rapid implementation, and lack of consultation and preparation. These findings concur with those of Eve *et al*.[44], Miller *et al*. [45] and Dunning *et al*. [46], who found that establishing effective communication, securing local ownership through consultation, and providing training opportunities for staff were vital for the successful implementation of practice change.

The change management strategy needed to build on positive prior experience by clarifying and establishing lines of communication with key individuals and groups across all organisational levels, and fostering local ownership by engaging in face-to-face meetings with targeted clinical leaders and professional teams. The use of a training intervention to implement new stroke assessment needed careful consideration. Existing organisational commitment to continuing professional education strengthened the case for such an educational intervention, an approach supported in a recent systematic review [13]. The organisation was also committed to evidence-based medicine, clinical effectiveness, multidisciplinary working, and training in research and development methodologies, including critical appraisal. These findings indicated that development of evidence-based guidelines for stroke assessment, and utilising a multiprofessional guideline development group for critical appraisal could be considered as part of a tailored strategy.

Instability due to organisational restructuring is known to create a major barrier for change [43]. This constituted a major potential challenge for change management in this project, because it would be difficult to ameliorate or remove sources of instability. Possible facilitators which could offset the potential for instability were the very positive views of staff relating to the work environment, the current management structure, and explicit local support for the project. Building coalitions and partnerships with key stakeholders, setting up a steering group with influential support, and working across organisational boundaries as the merger evolved, would all be vital components of the project leader's role in attempting to manage and sustain change.

Another barrier, with the potential to cause discontinuity in stroke assessment, was the shortage of permanent nursing staff (leading to increased use of agency nurses) and occupational therapists (leading to prioritisation of home assessments). These workforce shortages reflected national recruitment problems, had been found to constrain change in other studies [13], and were unlikely to be resolved within the timescale for the current project. Although the presence of a stable core of experienced professional staff might help to offset this barrier, strategies would have to be implemented to involve agency nurses in any training required for stroke assessment and recording. The systematic reviews by Thomson *et al*. [7] emphasise the value of educational outreach in implementing change. Providing ward-based training for agency nurses on a day-to-day basis therefore offered one solution. However, this would have implications for the role of the project leader. Staff shortages and workloads also suggested that the timetable and delivery of an educational intervention would have to be flexible and delivered on a number of occasions to enable attendance.

Weaknesses in team climate in medicine and some areas of nursing, the prevalence of a restricted, unidisciplinary team concept, and concerns about efficacy of liaison in selected areas were clear barriers to the development and implementation of a multiprofessional assessment and recording system. Offsetting these were the strongly developed team climate in therapy and other nursing teams linked to the positive experiences of multidisciplinary meetings and liaison in other areas. Clearly, positive team role models existed within the organisation and could be supportive of future change. The involvement of professional groups and support of multidisciplinary team development to secure a common understanding and commitment have been acknowledged as important in supporting change in other studies [43,46]. Implications for this strategy were that a common focus for teamwork could be created within a multidisciplinary guideline group developing evidence-based assessments, and in the delivery of a shared, workshop-based education programme that fostered interaction and utilised role play based on clinical practice. Multiprofessional education can be of benefit where it is interactive and relates to the reality of practice; professional education delivered using workshop-based approaches is also more likely to be effective in implementing change [47,48].

In relation to the current status of stroke assessment and recording, the main diagnostic findings were that assessment was fragmented, unidisciplinary, and lacked cohesion; recording practices did not facilitate information transfer between staff or, on discharge, to patients. Although assessment was, in part, evidence-based, the information used was narrow in range and did not reflect the scope offered by published evidence [49]. Moreover, information was not sourced from evidence-based guidelines. Implications for the development of a strategy for change were that a multidisciplinary advisory group could undertake a review of the evidence to support the development of a new assessment and recording system. The strong support within the organisation for critical appraisal training could assist this, but it was recognised that the project leader would also need to facilitate the work of the guideline development group.

Effective leadership is intrinsic to the success of a change management project [46,50,51] and the diagnostic findings had implications for the developing role of the project leader in this study. The scope of the project leader's role would encompass building organisational support at all levels, establishing local ownership, communication networks, and working across organisational boundaries – all recognised as key elements of partnership working to support change [21]. In addition, support for an educational intervention encompassing outreach and facilitation of multidisciplinary teamwork would demand a range of change agent skills in role modelling, establishing credibility, using facilitation, negotiation, participation, and critical appraisal to influence the uptake of change. These requirements for leadership are consistent with the role of opinion leaders, and are shown in some studies to benefit changes in practice [7]. In fulfilling this role, the project leader would need to draw support from clinical leaders representing each professional discipline to champion the uptake of change.

Diagnostic analysis is underpinned by the stage models of change which emphasise that change occurs in predictable linear phases. Implementation theory supports this view and in addition recommends organisational diagnosis and planned change [52]. However, there is a challenge to this approach from complexity theory which suggests that change cannot be planned, but instead occurs spontaneously. It suggests that complex organisations are living systems that co-evolve with their environment. Furthermore, organisations cannot be reduced to their component parts, but instead need to be viewed holistically [53]. Complexity theory suggests complex organisations exhibit non-linear behaviour that is unpredictably linked to input. This complex behaviour sits somewhere between predictability and non-predictability [54], and is recognised as on the 'edge of chaos' [55]. All of this suggests that change cannot be planned or organised, but rather it supports spontaneous change that evolves with little or no management contribution. This therefore raises issues regarding the usefulness of diagnosing organisations and planning change. Within the framework of complexity theory, diagnostic analysis would be a fluid rather than a fixed entity to accommodate and reflect the instability of organisations. Furthermore, the snapshot nature of data collection in diagnostic analysis would give way to data collection at more than one time point to capture movement in the organisation.

The theoretical framework underpinning this diagnostic analysis is broad and inclusive of competing theories. This provided a robust framework that could be useful not only as a precursor to change management in stroke care but also generalisable to other healthcare settings. The use of a theoretical framework to underpin the diagnostic analysis has resulted in the collection of a far broader range of data than if a purely empirical approach had been taken.

The broad range of findings identified from this diagnostic analysis highlights the usefulness of the approach for identifying barriers and facilitators prior to the implementation of change in clinical practice. Such an approach is recommended to inform the tailoring of implementation strategies to the specific organisational context. The implications are that a combination of qualitative and quantitative methods comprising interviews, documentary analysis, and a questionnaire to identify team climate can be used to collect data to identify the barriers and facilitators to change. Furthermore, such a broad approach to data collection gives a range of key stakeholders the opportunity to contribute their opinions, perspectives, and raise any concerns. This is vital because it is these key stakeholders who will need to change their practice and support others to do so. The more far-reaching implications of this approach can be learned only in hindsight once the findings have been used to inform the change management strategy and the outcome has been evaluated.

## Conclusion

Diagnostic analysis had been recommended as a precursor to change; however, few specific methodological approaches had been proposed. This study proposed the use of a combination of qualitative and quantitative methods to include analysis of corporate documents, interviews with staff and patients, and a survey of team climate. The use of multiple methods ensured that a range of data and respondents were included. The findings supported the use of leadership, evidence-based guidelines for assessment linked to a new recording system, and education as part of a combined strategy to implement changes in local stroke assessment practices.

## Competing interests

The author(s) declare that they have no competing interests.

## Authors' contributions

SH contributed to the study design, undertook data collection, data analysis and wrote the first draft of the paper and subsequent revision. SMcL developed the study concept and design, contributed to data collection, and made a major contribution to analysis and revision of the paper. AM advised on study design and made a major contribution to data analysis and interpretation. All authors read and approved the final manuscript.
